# Insane of Iowa

**Published:** 1854-04

**Authors:** 


### THE INSANE OF IOWA.

Some time since a Circular was addressed to certain officers of the different counties, signed by Judges Lowe and Johnson of this county, and one of the members of the Medical Faculty of this place, declaring their conviction that there was an error in the returns made, in the last census in the state of the number of the insane. The object was to ascertain the facts which, when elicited, would have an important bearing upon future legislative action in our own State and aid materially the efforts now making in Congress for a grant of land for the establishment and maintainance of asylums. Notwithstanding the importance of the subject, we have only heard from twenty counties, and these by no means the most populous, and yet these counties, about the third only of the whole number, give about 40 insane, the number reported for the whole State. The counties of V. Buren, Desmoines and Lee and several other densely populated counties, are not included in the returns. We are glad to know from the Secretary of State that he has made it imperative upon certain officers in listing property, also to report the Insane, Blind, and Deaf and Dumb.

